# Adipose-derived autotaxin regulates inflammation and steatosis associated with diet-induced obesity

**DOI:** 10.1371/journal.pone.0208099

**Published:** 2019-02-07

**Authors:** J. Anthony Brandon, Maria Kraemer, Julia Vandra, Suchismita Halder, Margo Ubele, Andrew J. Morris, Susan S. Smyth

**Affiliations:** 1 Division of Cardiovascular Medicine, The Gill Heart and Vascular Institute, University of Kentucky, Lexington, KY, United States of America; 2 Department of Veterans Affairs Medical Center, Lexington, Kentucky, United States of America; State University of Rio de Janeiro, BRAZIL

## Abstract

Autotaxin (ATX) is a secreted enzyme that generates the bioactive lipid lysophosphatidic acid (LPA). We generated mice with global inducible post-natal inactivation or adipose-specific loss of the *Enpp2* gene encoding ATX. The animals are phenotypically unremarkable and exhibit differences in adipocyte size and adipose tissue expression of inflammatory genes after high fat feeding without gross differences in fat distribution or body mass. Surprisingly, both models of *Enpp2*- deficiency exhibited marked protection from high fat diet-induced hepatic steatosis. This phenotype was not associated with differences in dietary fat absorption but may be accounted for by differences in hepatic expression of genes involved in *de novo* synthesis of triglycerides. These findings suggest that pharmacological inhibition of ATX might be protective against hepatic steatosis.

## Introduction

The ectonucleotide pyrophosphatase /phosphodiesterase 2 (ENPP2), also referred to as autotaxin (ATX), is a secreted lysophospholipase D (lysoPLD) that catalyzes the hydrolysis of circulating or cell-associated lysophosphatidylcholine (LPC) to generate the bioactive lipid mediator lysophosphatidic acid (LPA). ATX was originally identified as cell motility factor for cancer cells [1], has an identified role in vascular development [2–4], and serves as a marker and potential mediator of fibrosis [5–7] and inflammation [8–11]. Most, if not all, of its biologic effects appear to be mediated by the generation of LPA, which has broad ranging and potent, receptor-mediated effects on cells. Indeed, LPA promotes cell growth, differentiation, apoptosis and development [12, 13], largely through effects on a family of G-protein-coupled LPA receptors with at least six bona fide members (LPA1-6 receptors) and potentially also through the immunoglobulin family receptor for advanced glycan end products (RAGE) [14]. ATX can also bind cell surface receptors, such as integrins [15–18], raising the possibility of LPA-independent effects on cell adhesion and motility.

ATX contains a consensus signal sequence, which is expressed as a transmembrane protein and after processing and secretion undergoes classic processing for extracellular secretion, and is found in most biologic fluids, including plasma. Multiple cells secrete ATX, with adipocytes contributing substantially to levels in circulation [19, 20]. Adipocytes express and secrete ATX during differentiation [21], in a manner that requires gp130 [22]. Both visceral and subcutaneous fat contain mRNA for ATX, and the expression is largely restricted to adipocytes with little to no expression observed in stromal vascular cells (preadipocytes, mesenchymal stem cells, endothelial progenitor cell, T cells, B cells, mast cells and adipose tissue macrophage). AP2 Cre-specific ablation of the *Enpp2* gene lowers ATX concentrations in plasma [20], suggesting that adipocytes are an important source of circulating ATX. In keeping with these observations and the role of ATX and LPA in inflammation, the enzyme has been proposed as a novel adipokine. However, its precise function in the context of obesity is not clear. Some have reported that with obesity, ATX expression increases particularly in subcutaneous fat depots [19] and others have suggested that ATX mRNA levels are higher in adipose from individuals with insulin resistance [23]. However, there are reports that adipose ATX expression decreases in obese subjects [24]. Finally, plasma LPA and ATX levels may positively correlate with body mass index [21, 23, 25]. A similar degree of controversy exists in rodents. ATX mRNA levels are higher in adipose tissue of obese db/db mice. We previously demonstrated that transgene-driven overexpression of ATX promotes adiposity and hepatic steatosis in mice [26]. However, there have been conflicting reports about whether reductions in ATX levels protect [24] or promote [20] adipose tissue expansion in obese mice. To reconcile these observations and to determine if adipose-derived ATX serves as a novel inflammatory mediator of obesity, we compared the phenotype of mice with systemic reductions in ATX levels postnatally with mice lacking adipose-derived ATX. Our findings suggest that ATX promotes local tissue inflammation and that adipose-derived ATX fundamentally regulates steatosis and lipid remodeling within liver in the context of diet-induced obesity without gross effects on adipose expansion and body weight.

## Materials and methods

### Animals

All procedures conformed to the recommendations of “Guide for the Care and Use of Laboratory Animals” [27] and were approved by the Institutional Animal Care and Use Committee at the University of Kentucky. Mice are monitored daily by trained veterinary staff and animal facility personnel for pain and discomfort. Any mice showing signs of distress that fail to respond to treatments recommended by a veterinarian were promptly euthanized. Systemic post-natal reduction in ATX expression was achieved by crossing mice containing loxP sites flanking exons 3 and 4 of *Enpp2* (fl/fl; previously backcrossed to C57BL/6) with B6.Cg-Tg(Mx1-cre; Jackson Laboratories) mice expressing Cre-recombinase under the control of the MX-1 promoter. At postnatal day 3–5, littermate fl/fl mice with and without the MX1-Cre were treated with polyinosinic:polycytidylic acid (pI-pC), which activates MX-1 to drive Cre expression and globally reduce ATX expression to generate MX1-Δ animals. ATX expression in adipocytes was targeted by crossing fl/fl mice with B6;FVB-Tg(Adipoq-cre; Jackson Laboratories) mice expressing Cre under the control of the adiponectin promoter (Adipoq). Mice were genotyped and weaned at 21 d, maintained on a 14-h light and 10-h dark cycle. At 5–6 weeks of age, littermates were assigned to either normal chow (2018 Teklad 18% rodent diet, Envigo, 2018C) or high fat diet (HFD; 60% of calories are from lard fat; Research Diet, D12492) and fed the diet a*d-libitum* for 20 weeks. Mice were euthanized using isoflurane overdose. Organs were isolated, weighed, and washed once with PBS prior to processing. An EchoMRI-100 Whole Body Composition Analyzer (University of Kentucky, Lexington, KY) was used to measure total fat and lean mass as previously described [26].

### Plasma cholesterol, triglyceride, ATX activity, and biomarkers

Total plasma cholesterol was measured using the Wako Diagnostics Cholesterol E Assay and triglycerides (TG) using the Wako Diagnostics L-type TG M Assay and the Multi-Calibrator Lipid Standard to generate a standard curve according to the manufacturer’s protocol. ATX activity was measured by choline release assay [28] in which choline is oxidized to betaine and hydrogen peroxide, the latter reacts with TOOS (N-ethyl- N-(2-hydroxy-3-sulfopropyl)-3 methylaniline) and 4-AAP (aminoantipyrene) to form quinoneimine dye which can be measured at Abs 555 nm every 5 minutes for 20 minutes. To establish background choline levels, a potent ATX inhibitor was included in triplicate samples. For each sample, the absorbance was plotted against time and the slope (dA/min) was calculated for the linear (steady-state) portion of each reaction. ATX activity was calculated according to the following equation: Activity (U/ ml) = (μmol/ min/ ml) = [dA/ min (sample)—dA/ min (blank)] * Vt/ (e* Vs* 0.5) where Vt: total volume of reaction (ml), Vs: volume of sample (ml), e: milimolar extinction coefficient of quinoneimine dye under the assay conditions (e = 32,8 μmol/ cm^2^) and 0.5: the moles of quinoneimine dye produced by 1 mol of H_2_O_2_. Immunoblotting for ATX was performed with a polyclonal antibody (AB#10005375, Cayman Chemicals). Plasma biomarkers were measured in EDTA-anticoagulated blood by Multiplex assay.

### Glucose, insulin, and lipid tolerance testing

Intraperitoneal glucose tolerance test was performed in the afternoon after a 5-h fasting. After baseline glucose measurements were taken, mice were injected i.p. with glucose (2 g/kg body weight) in isotonic saline, and serial measurements of glucose level were taken at 7, 15, 30, and 120 min. Intraperitoneal insulin tolerance test was performed in the afternoon after a 5-h fasting. After baseline glucose measurements were taken, mice were injected i.p. with insulin (2 g/kg body weight) in isotonic saline, and serial measurements of glucose level were taken at time points up to 120 min. Dietary fat absorption was measured using a modification of a previously described method [29] that involves feeding mice diets containing poorly absorbed sucrose fatty acid esters that can be measured in feces along with other diet derived fatty acids. Normalized fat absorption can then be calculated from the ratio of free and esterified fatty acids in the diet and feces to the sucrose fatty acid ester measured in the same samples after measurement by GC MS.

### Histology

For adipocyte cell size, slides containing frozen fat tissue sections (subcutaneous and visceral) were stained with BODIPY dye. Cell area determined after imaging on a Nikon A1R confocal microscope with a spectral detector and analysis performed using NIKON NIS software (Melville, NY) where 100 cells/tissue/mouse were measured.

### Quantitative Real-time PCR

Organs were collected from mice and weighed. A representative piece from taken from similar locations in each organ was immediately cut and stored in RNAlater (Thermo Fisher Scientific, Waltham, MA, Ca# AM7021). The tissues were homogenized and total RNA was extracted using Trizol (Thermo Fisher Scientific, cat# 15596018). cDNA was prepared with Multiscribe reverse-trasncriptase enzyme (High Capacity cDNA Archive Kit; Thermo Fisher Scientific). mRNA expression was measured in real-time quantitative PCR using predesigned TaqMan gene expression assays (Thermo Fisher Scientific) as indicated in S1 Table. All the probes used in the study spanned an exon junction and thus would not detect genomic DNA. An RNA sample without reverse transcription was used as a negative control. Samples were assayed on a Quantstudio 7 flex (Applied Biosystems). Threshold cycles (CT) were determined by an in-program algorithm assigning a fluorescence baseline based on readings prior to exponential amplification. Fold change in expression was calculated using the 2-δδCT method using 18s RNA as an endogenous control.

### Lipidomics

Lipids were extracted from tissue using acidified organic solvents with inclusion of lipid class specific internal standards. The lipid containing organic phase was evaporated to dryness, reconstituted and analyzed by direct infusion mass spectrometry using an AB Sciex 5600 (Sciex, Framingham, MA) quadrupole time of flight mass spectrometer and adaptations of previously described methods [30, 31]. In brief data were collected in positive and negative mode with collection of a survey scan followed by sequential precursor ion selection and fragmentation at unit mass increments in the range m/z 200–1,200. The data were processed using AB Sciex LipidView software (Sciex) with a mass tolerance set to 0.3 mDa and spectral peaks exceeding 2 times the signal to noise threshold included. The software identifies lipids from their accurate molecular ion mass, isotope pattern and product ion spectra. After inspection of the primary data, lipid species detected in all technical replicates were quantitated by reference to their cognate lipid class specific internal standard. Although these experiments necessarily identify a large number of molecular species within each lipid class for simplicity the data presented are a sum of all lipid species within a particular class normalized to the starting weight of the tissue.

### Statistical analysis

All results are expressed as means ± SD. Statistical significance within strains was determined using a Student's t-test or two-way ANOVA with multiple pairwise comparisons as appropriate. In t-tests, if a sample failed the normality test, a rank t-test was used. In some cases of two-way ANOVA, data were log-transformed to be normally distributed. Statistical analysis was performed using Sigma-plot 13 software. A value of P < 0.05 was considered significant.

## Results

To define a role for ATX in obesity and its complications, two animal models with reduced ATX levels were established. Germline inactivation of the *Enpp2* gene, encoding ATX, is embryonically lethal, therefore systemic ATX reduction was achieved postnatally by breeding mice with exons 3 and 4 of *Enpp2* flanked by lox-P sites (fl/fl) to mice carrying the Cre recombinase under the control of the MX-1 promoter and activating the promoter in neonatal pups with synthetic double-stranded RNA (MX1-Δ mice). Adipocyte-specific loss of ATX was achieved with Cre recombinase under the control of the adiponectin promoter (Adipoq-Δ mice). To elicit diet-induced obesity, littermate mice with or without the Cre recombinase transgene were placed on HFD for 20 weeks.

**Fig 1:**
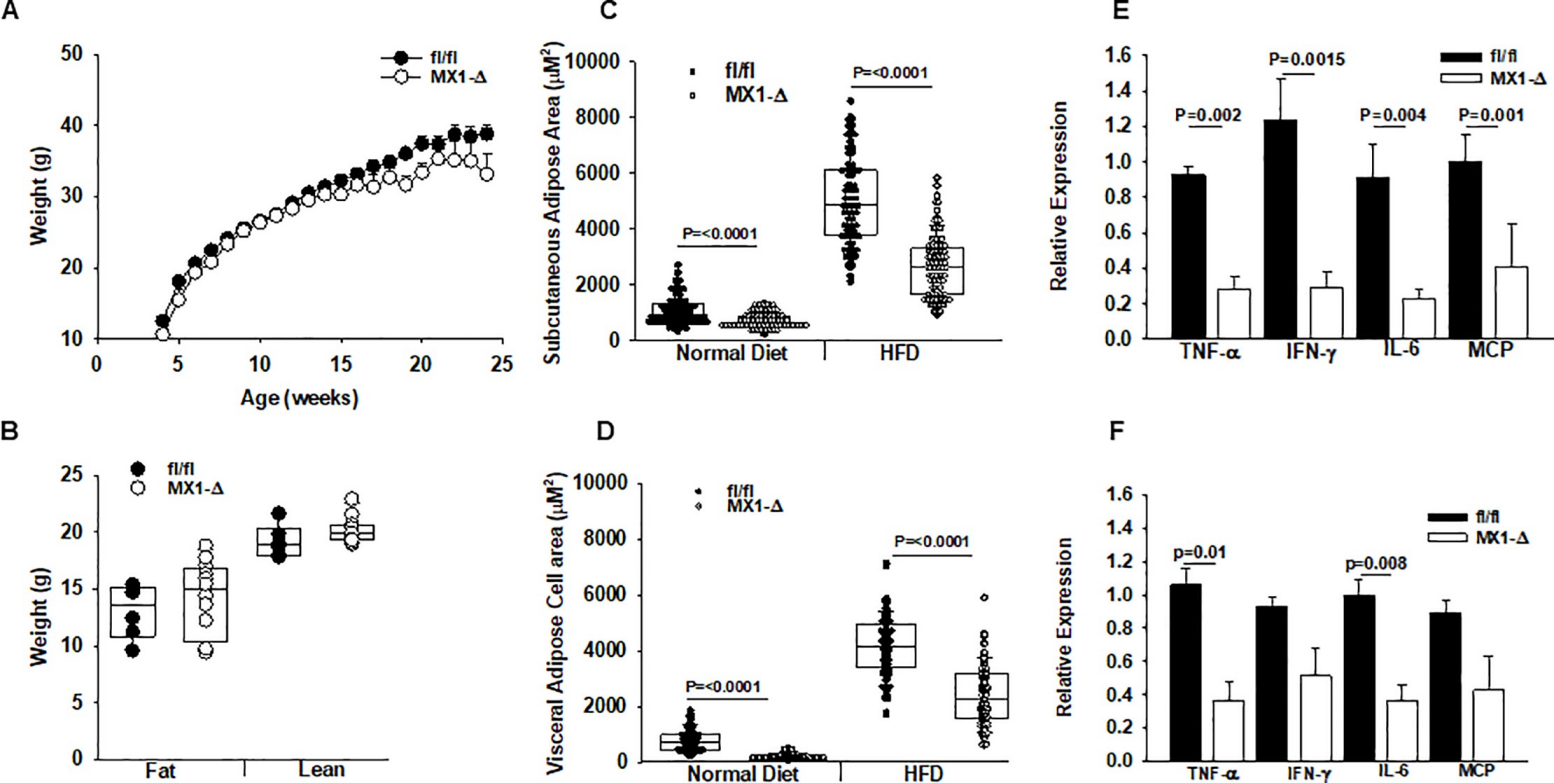
Post-natal reductions in ATX expression alters adipocyte size and adipose tissue inflammation without an effect on body weight after HFD. **A.)** Average body weight in fl/fl (dark circles) and MX1-Δ (open bars) male mice (n = 10-15/group at the indicated ages (weeks) after initiating HFD at 4 weeks of age. Mice were placed on HFD at 4 weeks of age. **B.)** Body composition by fat and lean weight in fl/fl (dark circles) and MX1-Δ (open bars) male mice (n = 8–15 mice/group) after 20 weeks on HFD. **C.)** Cell area in subcutaneous adipose tissue in fl/fl (dark circles) and MX1-Δ (open bars) male mice (n = 4 mice; 100 cells/mouse) on normal chow or after 20 weeks on HFD. **D.)** Cell area in visceral adipose tissue in fl/fl (dark circles) and MX1-Δ (open bars) male mice (n = 4 mice and 100 cells/mouse) on normal chow or after 20 weeks on HFD. **E.)** Gene expression in subcutaneous adipose tissue from fl/fl (dark circles) and MX1-Δ (open bars) male mice after 20 weeks on HFD (n = 3–4). **F.)** Inflammatory gene expression in visceral adipose tissue from fl/fl (dark circles) and MX1-Δ (open bars) male mice after 20 weeks on HFD (n = 3–4). Comparison between genotypes was performed by t-test. * = P<0.05.

Global reduction in *Enpp2* expression was observed in MX1-Δ mice (S1A Fig; open bars), with significantly lower levels of gene expression in kidney and spleen, and to a lesser extent in heart and lung as compared to identically treated fl/fl littermate controls (S1 Fig; dark bars). ATX activity was reduced by approximately 50% in plasma from MX1-Δ mice (S1B Fig). ATX protein in subcutaneous and visceral adipose tissue was also lower in MX1-Δ as compared to fl/fl controls (S1C Fig). Together, these results confirm the effectiveness of the post-natal strategy to reduce *Enpp2* expression and ATX levels. No significant difference was observed between the MX1-Δ and fl/fl mice in weight gain on HFD (Fig 1A) or fat and lean mass after 20 weeks of diet (Fig 1B) or adiposity index (13.2 ± 1.1 and 10.7 ± 4, n = 13; P = 0.43). Plasma TG (42.2 ± 10.9 and 49.6 ± 31 mg/dl), non-esterified fatty acids (7.1 ± 1.8 and 8.9 ± 3.5 mg/dl) or phospholipids (42.9 ± 8.0 and 60.5 ± 6 mg/dl) were similar in fl/fl and MX1-Δ mice (mean ± SD; n = 4–6). No difference in heart, liver, lung or spleen weight was observed. Interestingly, adipocyte cell size in both subcutaneous (Fig 1C) and visceral (Fig 1D) fat was significantly smaller in the MX1-Δ mice. In parallel with the changes in cell size, inflammatory marker expression in subcutaneous (Fig 1E) and visceral (Fig 1F) adipose tissue was also lower in MX1-Δ animals. In particular, gene expression for tumor necrosis factor alpha (*Tnfα*, interferon gamma (*Ifnγ*, interleukin-6 ***(****IL-6)* and chemokine CC motif ligand 2 (*Mcp1)* were all reduced in the MX1-Δ mice. These findings suggest a role for ATX in regulating adipocyte size and inflammatory gene expression during high fat feeding.

**Fig 2:**
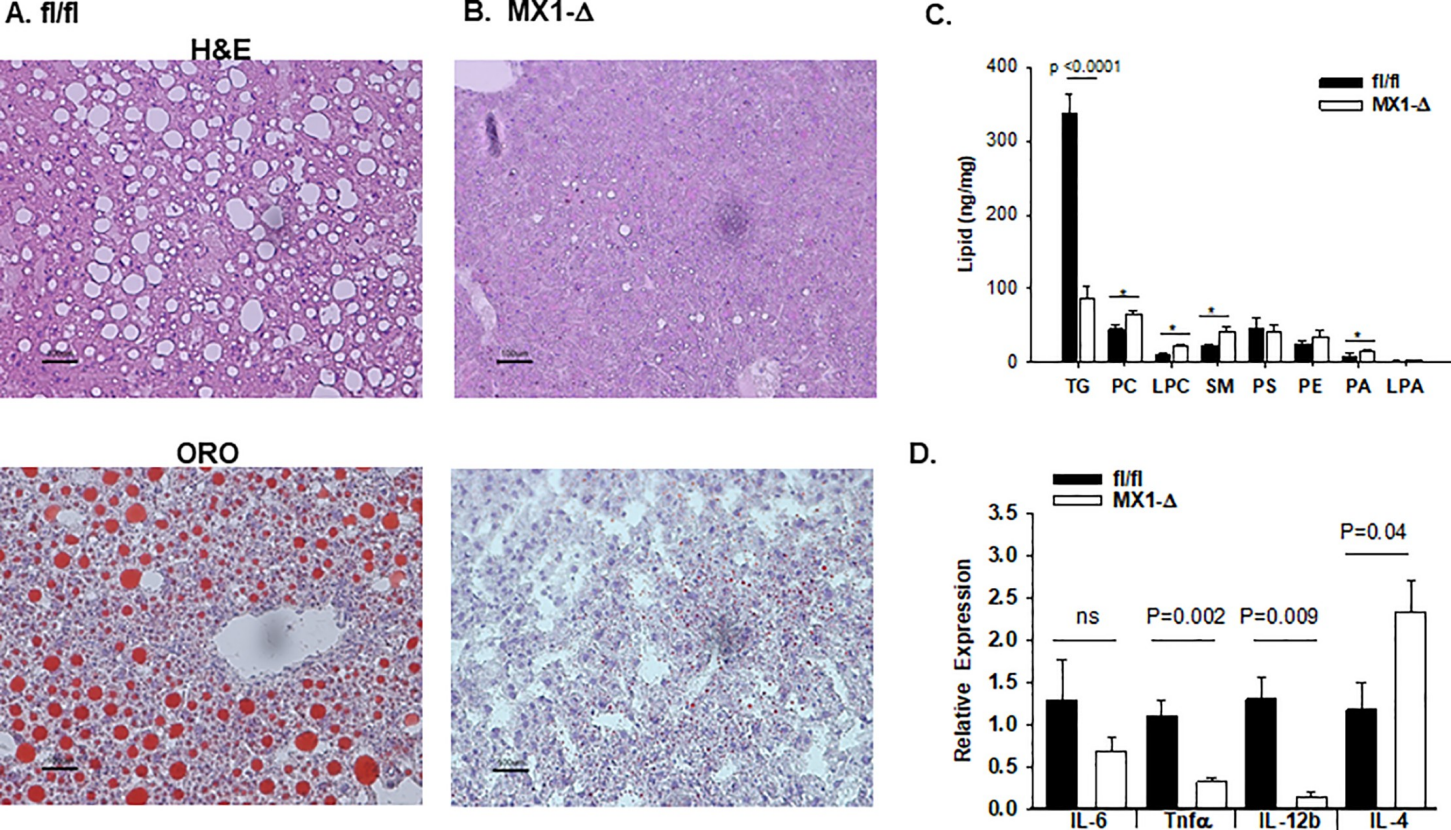
Post-natal reduction in ATX expression protect from diet-induced changes in hepatic lipid accumulation. **A.)** Representative images (20X) of liver sections stained with H&E (top) and ORO (bottom) from fl/fl mice after 20 weeks on HFD. **B.)** Representative images (20X) of liver sections stained with H&E (top) and ORO (bottom) from MX1-Δ mice after 20 weeks on HFD. **C.)** Lipid profiles from liver of fl/fl (dark bars) and MX1-Δ (open bars) male mice after 20 weeks on HFD. Summed results for indicated lipid species are displayed (n = 4/group). **D.)** Inflammatory gene expression in liver from fl/fl (dark bars) and MX1-Δ (open bars) male mice after 20 weeks on HFD (n = 3). Comparison between genotypes was performed by t-test. * = P<0.01.

When on HFD, mice accumulate ectopic lipid in non-adipose tissues such as liver, resulting in hepatic steatosis. We therefore investigated the impact of ATX deficiency on high fat diet induced lipid accumulation in different tissues and observed differences in hepatic lipid accumulation. Both hematoxolyin-eosin (H&E) and oil-red-O (ORO) staining for neutral lipid reveal macrovesicular lipid accumulation in fl/fl control mice (Fig 2A). In contrast, MX1-Δ mice were strikingly protected from neutral lipid accumulation as visualized by ORO staining (Fig 2B). Triglyceride levels were about 50% lower in liver from MX1-Δ mice (3.7 ± 2 versus 1.6 ± 0.5 per mg liver). Lipidomic profiling confirmed lower TG levels in livers from the MX1-Δ mice, and revealed statistically significant increases in phosphatidylcholine (PC), sphingomyelin (SM) and phosphatidic acid (PA) in mice with reduced ATX (Fig 2C). No significant differences in liver phosphatidylserine (PS) or phosphatidylethanolamine (PE) were observed. The PC/PE ratio was not statiscally different in the fl/fl versus MX1-Δ livers (1.86 ± 0.44 versus 1.94 ± 0.39; mean ± SD; n = 3; P = 0.82). No difference in LPA receptor expression was observe in livers (S2 Fig), and gene expression levels of several enzymes that regulate synthesis of cholesterol steroids and other lipids was largely unaffected by reduced ATX, with the exception of *Cyp8B1* (cytochrome P450, family 8, subfamily B, polypeptide 1) that regulates intestinal bile acid composition (S2 Fig). Along with alterations in lipid accumulation, substantially lower gene expression of key pro-inflammatory markers, *Tnfα* and *IL-12β*, was observed with an increase in *IL-4* expression in MX1-Δ liver (Fig 2D).

**Fig 3:**
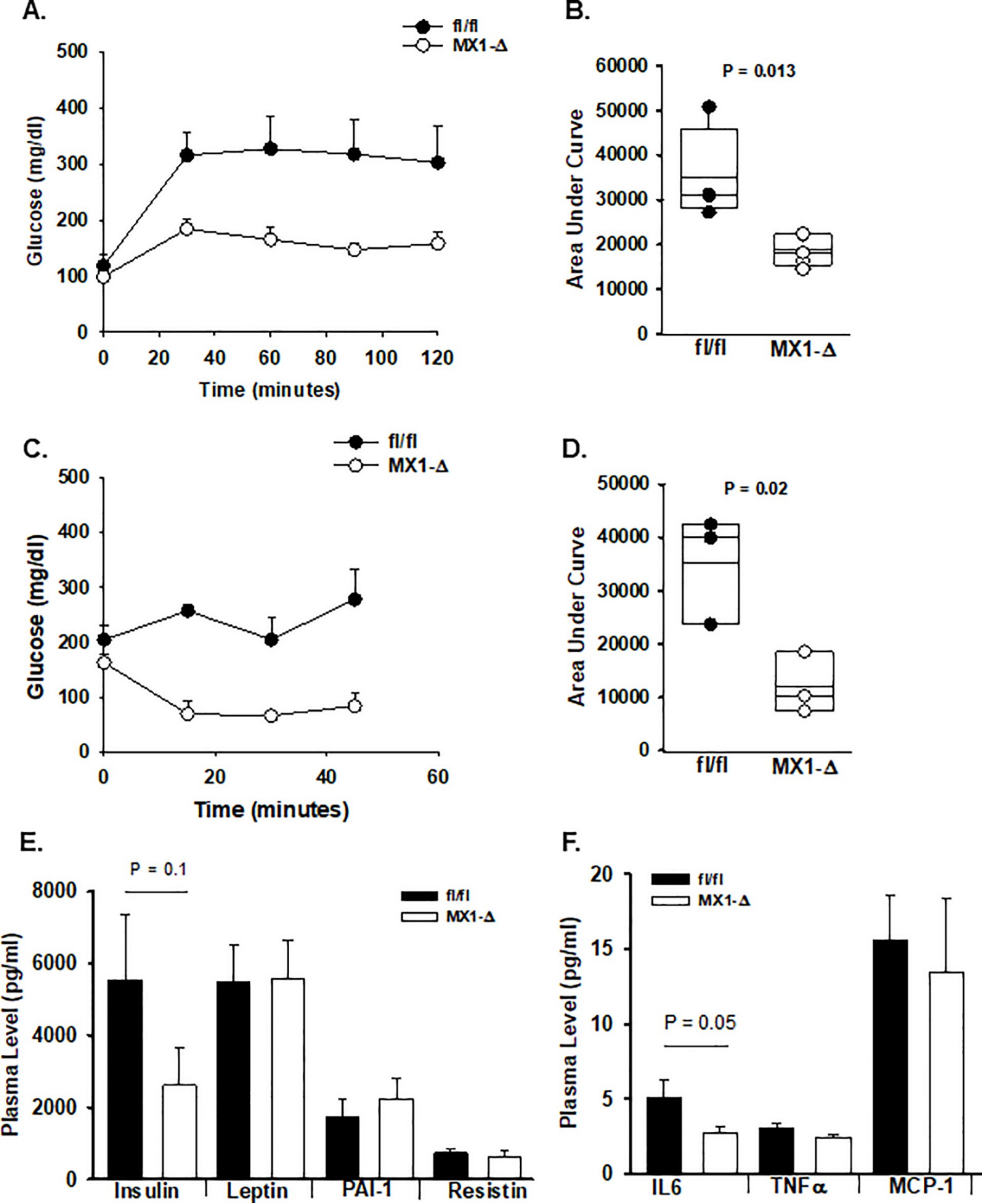
Post-natal reductions in ATX expression alters glucose tolerance. **A.)** After HFD, fl/fl (dark bars) and MX1-Δ (open bars) male mice were injected with glucose (2 g/kg body weight in isotonic saline, i.p.) and serial measurements of blood glucose levels were made at the indicated times. Results are presented as mean ± SD (n = 4–5 mice). **B.)** Area under the curve (AUC) for glucose tolerance for each individual animal was calculated and presented for individual mice. **C.)** Blood glucose levels at the indicated times after injection of insulin (1.5 U/kg body weight in isotonic saline, i.p.) (n = 3). **D.)** Area under the curve (AUC) for insulin tolerance for each individual animal was calculated and presented for individual mice. Box plots represent 25–75%iles; dark bar median. **E.)** Plasma levels (mean ±SD in pg/ml) of insulin, leptin, PAI-1, and resistin in fl/fl (dark bars) and MX1-Δ (open bars) male mice after HFD (n = 5). **F.)** Plasma levels (mean ±SD in pg/ml) of IL-6, TNFα, and MCP-1 in fl/fl (dark bars) and MX1-Δ (open bars) male mice after HFD (n = 6–7).

Hepatic steatosis is associated with hepatic insulin resistance. Therefore, the systemic impact of these changes was examined in fl/fl and MX1-Δ mice after 20 weeks on HFD. Baseline glucose levels were similar in mice of both genotypes, however, glucose levels were substantially higher after an intraperitoneal glucose challenge in the fl/fl mice (Fig 3A and 3B). To determine if these changes altered insulin sensitivity, mice were injected intraperitonealy with a dose of insulin that had minimal effect on glucose levels in fl/fl mice, but that caused a drop in plasma glucose in MX1-Δ mice (Fig 3C and 3D), suggesting a difference in insulin sensitivity. Systemic levels of insulin were lower in nonfasting MX1-Δ mice with no change in plasma leptin, PAI-1 or resistin levels (Fig 3E). In keeping with the reduced inflammation in both adipose and liver tissue, plasma IL-6 levels were lower in MX1-Δ mice, however, no differences in plasma TNFα, MCP-1 (Fig 1F) or adiponectin were found.

**Fig 4:**
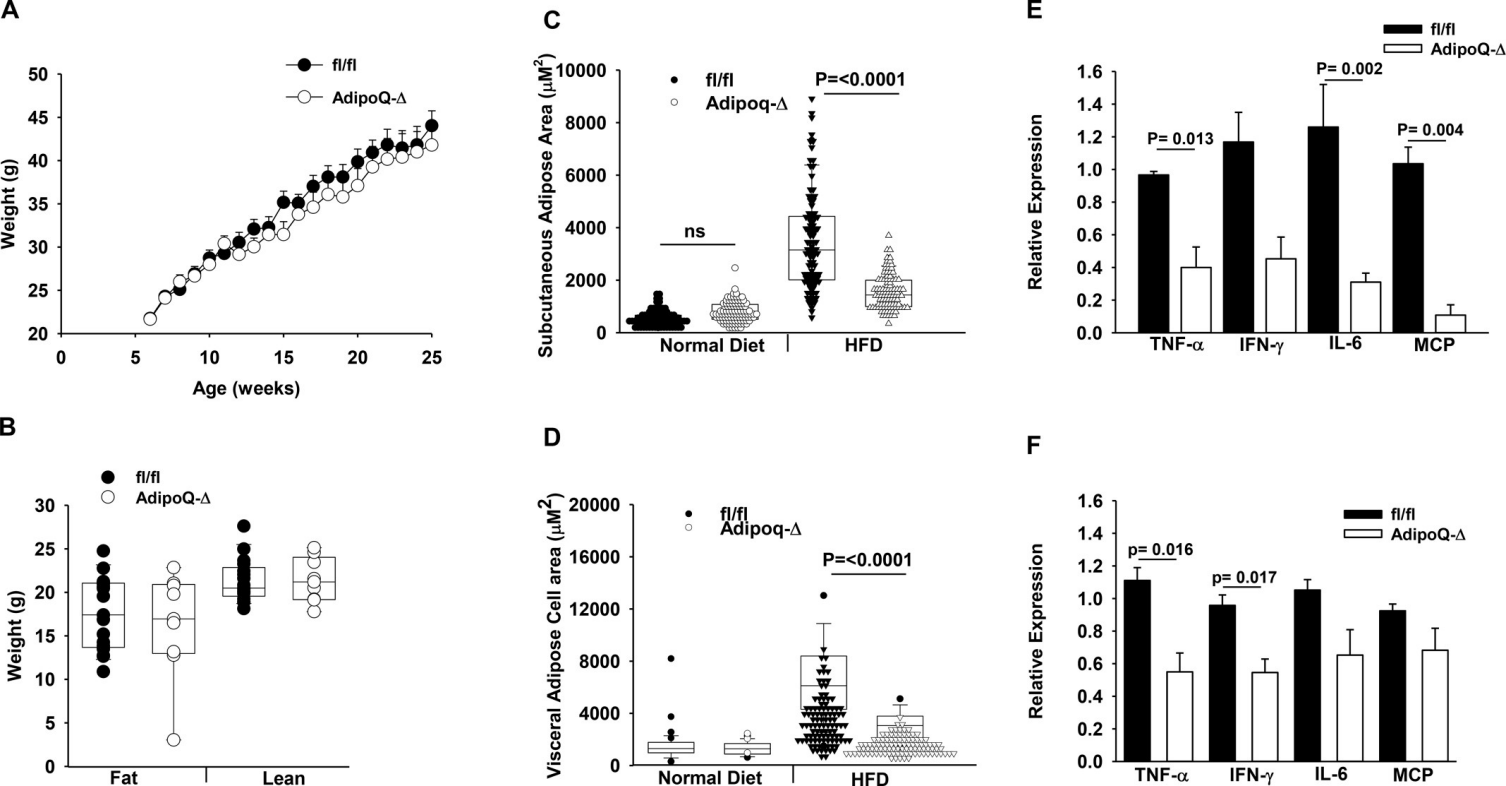
Loss of adipocyte ATX expression alters adipocyte size and adipose tissue inflammation without an effect on body weight after HFD. **A.)** Average body weight in fl/fl (dark circles) and Adipoq-Δ (open bars) male mice (n = 20/group) t the indicated ages (weeks) after initiating HFD at 4 weeks of age. Mice were placed on HFD at 4 weeks of age. **B.)** Body composition by fat and lean weight in fl/fl (dark circles) and Adipoq-Δ (open bars) male mice (n = 9–17) after 20 weeks on HFD. **C.)** Cell area in subcutaneous adipose tissue in fl/fl (dark circles) and Adipoq-Δ (open bars) male mice (n = 8) on normal chow or after 20 weeks on HFD. **D.)** Cell area in visceral adipose tissue in fl/fl (dark circles) and Adipoq-Δ (open bars) male mice (n = 8) on normal chow or after 20 weeks on HFD. **E.)** Gene expression in subcutaneous adipose tissue from fl/fl (dark circles) and Adipoq-Δ (open bars) male mice after 20 weeks on HFD (n = 3–4). **F.)** Inflammatory gene expression in visceral adipose tissue from fl/fl (dark circles) and Adipoq-Δ (open bars) male mice after 20 weeks on HFD (n = 3–4). Comparison between genotypes was performed by t-test. * = P<0.05.

To elucidate the contribution of adipose–derived ATX to lipid remodeling associated with obesity, the phenotype of the Adipoq-Δ mice fed HFD for 20 weeks was examined. No difference in kidney, spleen, lung, or heart *Enpp2* expression was observed between Adipoq-Δ mice and their fl/fl littermate controls (S3A Fig). ATX levels in subcutaneous fat were significantly reduced in Adipoq-Δ mice, as was plasma ATX activity (S3 Fig). No difference in weight gain over 20 weeks on HFD diet was noted between the two groups (Fig 4A), and lean and fat body masses were similar (Fig 4B) as was adiposity index (16.3 ± 1.3 and 15.8 ± 0.7, n = 6 and 13; P = 0.68). Plasma TG (47.9 ± 37 and 80 ± 59 mg/dl), non-esterified fatty acids (10.3 ± 2.5 and 11.6 ± 3.4 mg/dl) or phospholipids (147.4 ± 48.5 and 109 ± 38.2 mg/dl) were similar in fl/fl and MX1-Δ mice (mean ± SD; n = 4–10). Organs weights were also not different. As was observed with systemic ATX reduction, adipocyte-specific targeting of ATX resulted in smaller adipocyte size in mice fed HFD (Fig 4F and 4G), although not in mice on normal chow. Subcutaneous adipose (Fig 4H) and visceral adipose (Fig 4I) from Adipoq-Δ mice also displayed lower levels of some but not all inflammatory cytokine gene expression. In particular, gene expression for *Tnfα*, *IL-6*, and *Mcp-1* were lower in Adipoq-Δ subcutaneous fat (Fig 4E) and for *Tnfα* and *Ifnγ* in Adipoq-Δ visceral fat (Fig 4F).

**Fig 5:**
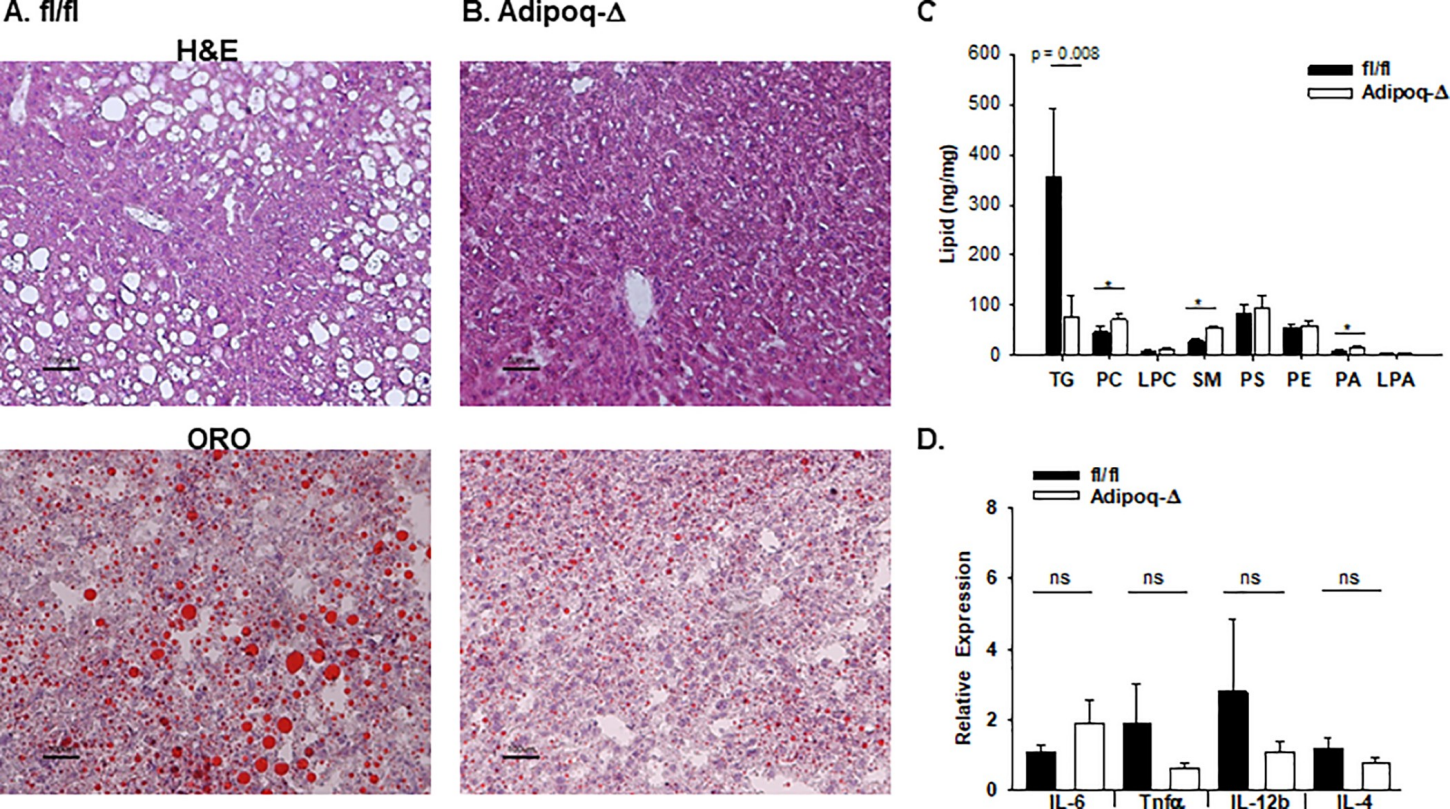
Loss of adipocyte ATX expression protects from diet-induced changes in hepatic lipid accumulation. **A.)** Representative images (20X) of liver sections stained with H&E (top) and ORO (bottom) from fl/fl mice after 20 weeks on HFD. **B.)** Representative images 20x) of liver sections stained with H&E (top) and ORO (bottom) from Adipoq-Δ mice after 20 weeks on HFD. **C.)** Lipid profiles from liver of fl/fl (dark bars; n = 4) and Adipoq-Δ (open bars; n = 4) male mice after 20 weeks on HFD. Summed results for indicated lipid species are displayed. **D**.) Inflammatory gene expression in liver from fl/fl (dark circles) and Adipoq-Δ (open bars) male mice after 20 weeks on HFD (n = 3). Comparison between genotypes was performed by t-test. * = P<0.01.

**Fig 6:**
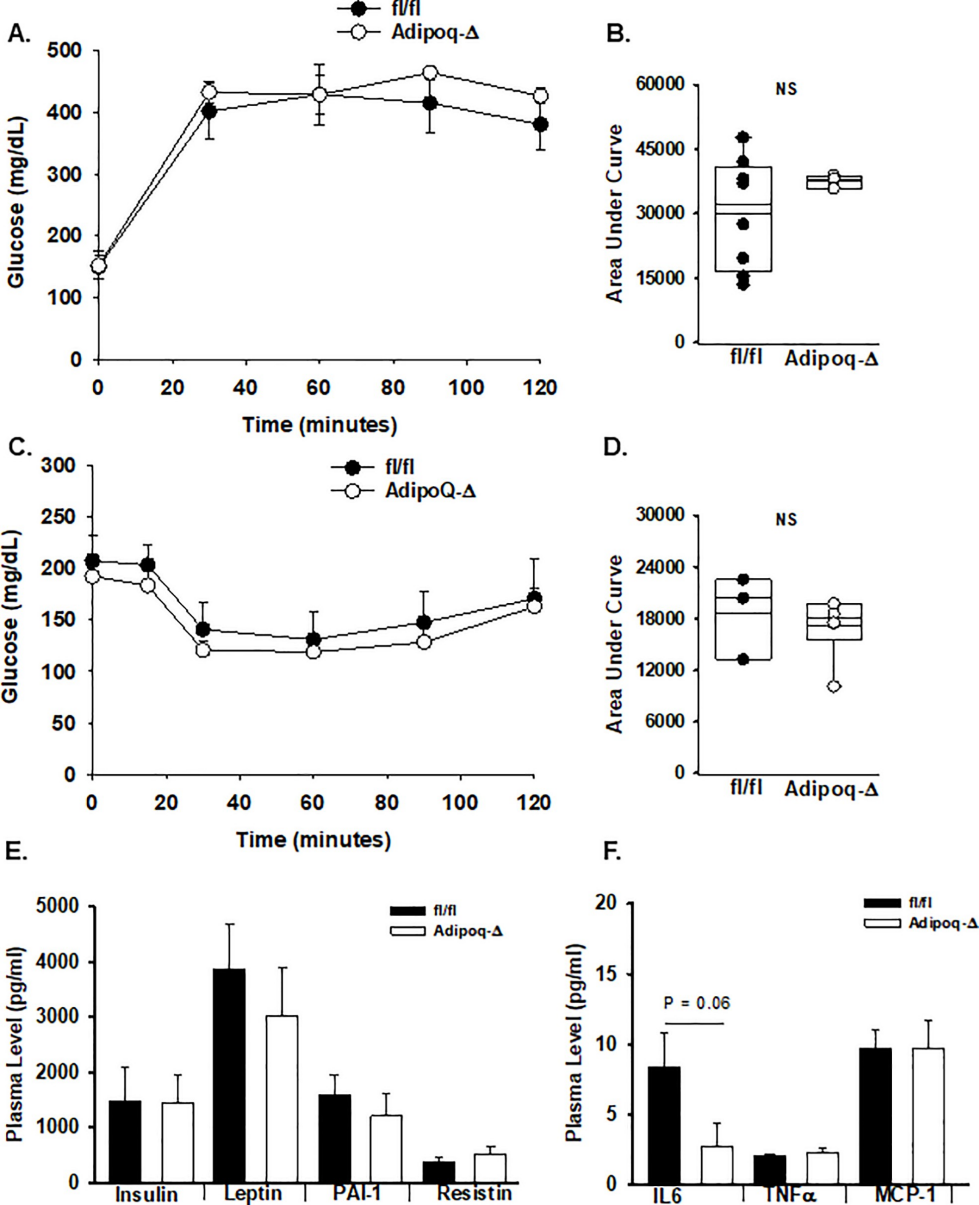
Loss of adipocyte ATX expression alter glucose tolerance. **A.)** After HFD, fl/fl (dark bars) and Adipoq-Δ (open bars) male mice were injected with glucose (2 g/kg body weight in isotonic saline, i.p.) and serial measurements of blood glucose levels were made at the indicated times. Results are presented as mean ± SD (n = 3–10). **B.)** Area under the curve (AUC) for glucose tolerance for each individual animal was calculated and presented for individual mice. Box plots represent 25–75%iles; dark bar median. **C.)** Blood glucose levels at the indicated times after injection of insulin (1.5 U/kg body weight in isotonic saline, i.p.) (n = 3–4). **D.)** Area under the curve (AUC) for insulin tolerance for each individual animal was calculated and presented for individual mice. Box plots represent 25–75%iles; dark bar median. **E.)** Plasma levels (mean ±SD in pg/ml) of insulin, leptin, PAI-1, and resistin in fl/fl (dark bars) and Adipoq-Δ (open bars) male mice after HFD. **F.)** Plasma levels (mean ±SD in pg/ml) of IL-6, TNFα, and MCP-1 in fl/fl (dark bars) and Adipoq-Δ (open bars) male mice after HFD (n = 7). Comparison between genotypes was performed by t-test. * = P<0.05.

As was observed with the MX1-Δ mice, liver histology confirmed less lipid accumulation in the Adipoq-Δ mice in comparison to their fl/fl littermate controls (Fig 5A and 5B) after HFD. Lipidomic profiling demonstrated significantly lower TG levels in livers from the Adipoq-Δ mice, along with statistically significant increases in phosphatidylcholine (PC), lysophosphatidylcholine (LPC), sphingomyelin (SM) and phosphatidic acid (PA) (Fig 5C). The PC/PE ratio was statistically higher in the Adipoq-Δ liver (1.2 ± 0.08 versus 0.84 ± 0.2; mean ± SD in fl/fl controls; n = 4; P = 0.036). No difference in LPA receptor or PPAR expression was observed (S4 Fig). Likewise, expression of inflammatory genes *IL-6*, *Tnfα*, *Mcp-1*, or *IL-4* (Fig 5D) was unchanged in Adipoq-Δ liver. In keeping with the lack of difference in inflammation, the Adipoq-Δ mice fed HFD displayed no difference in glucose (Fig 6A and 6B) or insulin tolerance (Fig 6C and 6D) in comparison to their littermate controls. Indeed, no difference was observed in plasma levels of insulin, leptin, PAI-1, or resitin (Fig 6E). No differences in plasma IL-6, TNFα or MCP-1 levels were observed between Adipoq-Δ and fl/fl mice after 20 weeks on HFD (Fig 6F), although IL-6 trended lower (P = 0.06).

**Fig 7:**
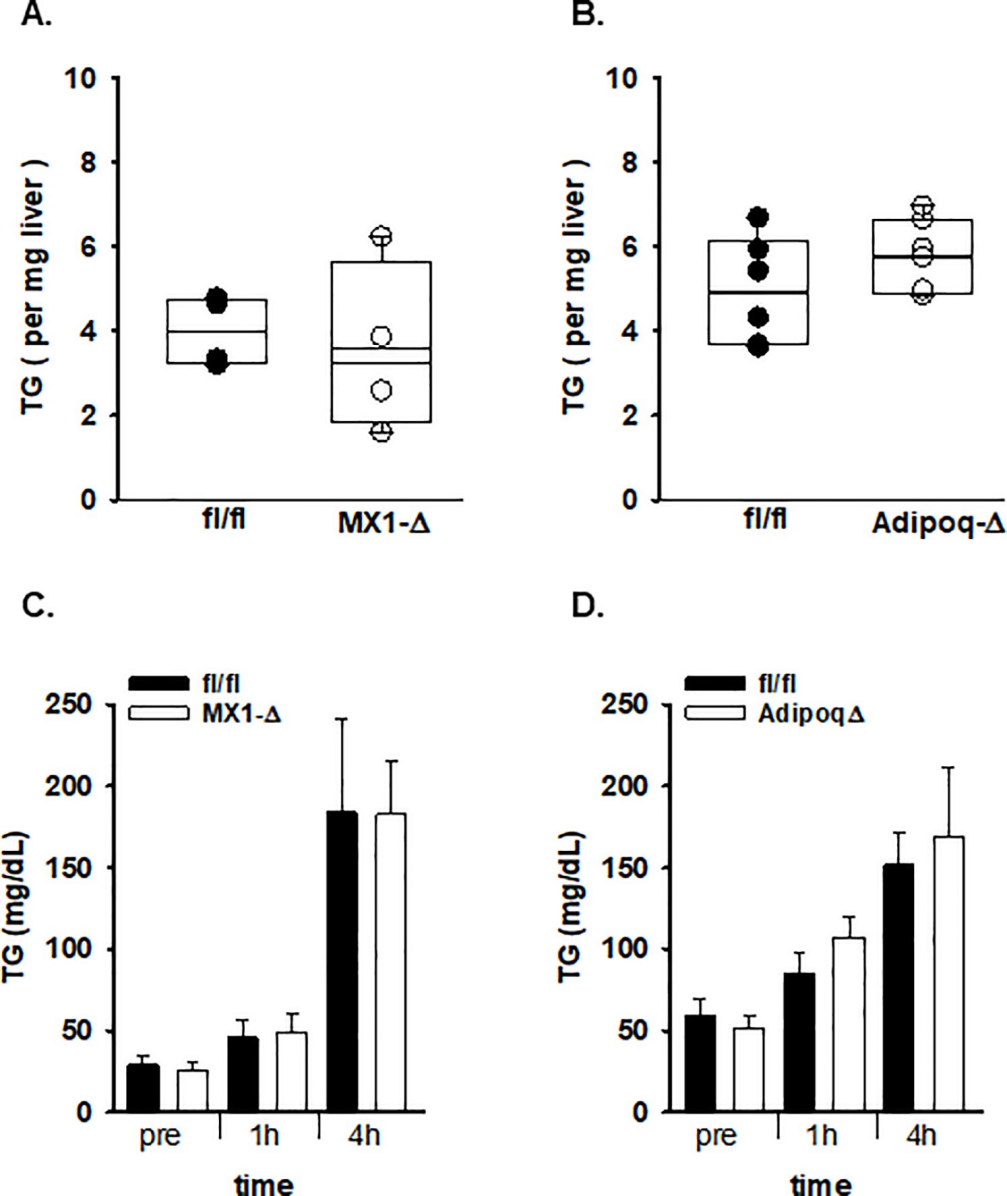
Lack of effect of ATX on TG accumulation in liver after fasting or in plasma after oral lipid challenge. **A.)** Liver TG levels after 24 hour fast in fl/fl (n = 4) and littermate control MX1-Δ (n = 4) male mice. Average values for individual mice; box plots indicated 25–75%iles and bar median. **B.)** Liver TG levels after 24 hour fast in fl/fl and littermate control Adipoq-Δ male mice (n = 6–7). **C.)** Plasma TG levels at the indicated time before (pre) or after oral lipid administration (250 μl olive oil by gavage) in fl/fl and MX1-Δ mice (n = 4). **D.)** Plasma TG levels at the indicated time before (pre) or after oral lipid administration (250 μl olive oil by gavage) in fl/fl and Adipoq-Δ mice (n = 3–10).

To understand the role of ATX in liver lipid remodeling, we sought to determine if the effect required dietary intake of high fat or was a general property observed in models of hepatic steatosis. In rodents, fasting increases hepatic TG as a result of re-esterification of free fatty acids that exceeds the liver’s capacity to secrete TG. We investigated the role of ATX in hepatic accumulation of TG as a consequence of fasting. As has been previously reported [32], twenty-four hours of fasting increased hepatic TG levels by approx. 3-fold from 1.6 ± 0.3 (n = 4) to 6.6 ± 1.7 (n = 7) per mg liver. No differences were observed between MX1-Δ and fl/fl littermate controls (Fig 7A) or between fl/fl and Adipoq-Δ mice (Fig 7B) in accumulation of hepatic TG after 24 hour fast, indicating the role of ATX in hepatic TG accumulation occurs in the context of dietary intake of fat. Therefore, we investigated whether ATX contributes to lipid absorption and handling by following administration of a bolus of lipid. Again, no differences in plasma TG levels after oral lipid administration in either MX1-Δ (Fig 7C) or Adipoq-Δ mice (Fig 7D) in comparison to their respective controls. In addition, we used a noninvasive method to measure intestinal fat absorption using diets containing poorly absorbed sucrose fatty acid esters to monitor intestinal absorption of dietary fat. Fat absorption was calculated from the ratios of sucrose fatty acid ester to other fatty acids in diet and feces as analyzed by mass spectrometry of fatty acid methyl esters. Using this method, no significant difference in fat absorption was observed between male fl/fl and Adipoq-Δ mice (ratio of 0.48 ± 0.02 and 0.49 ± 0.08, respectively) on day 4 after SPB diet.

**Fig 8:**
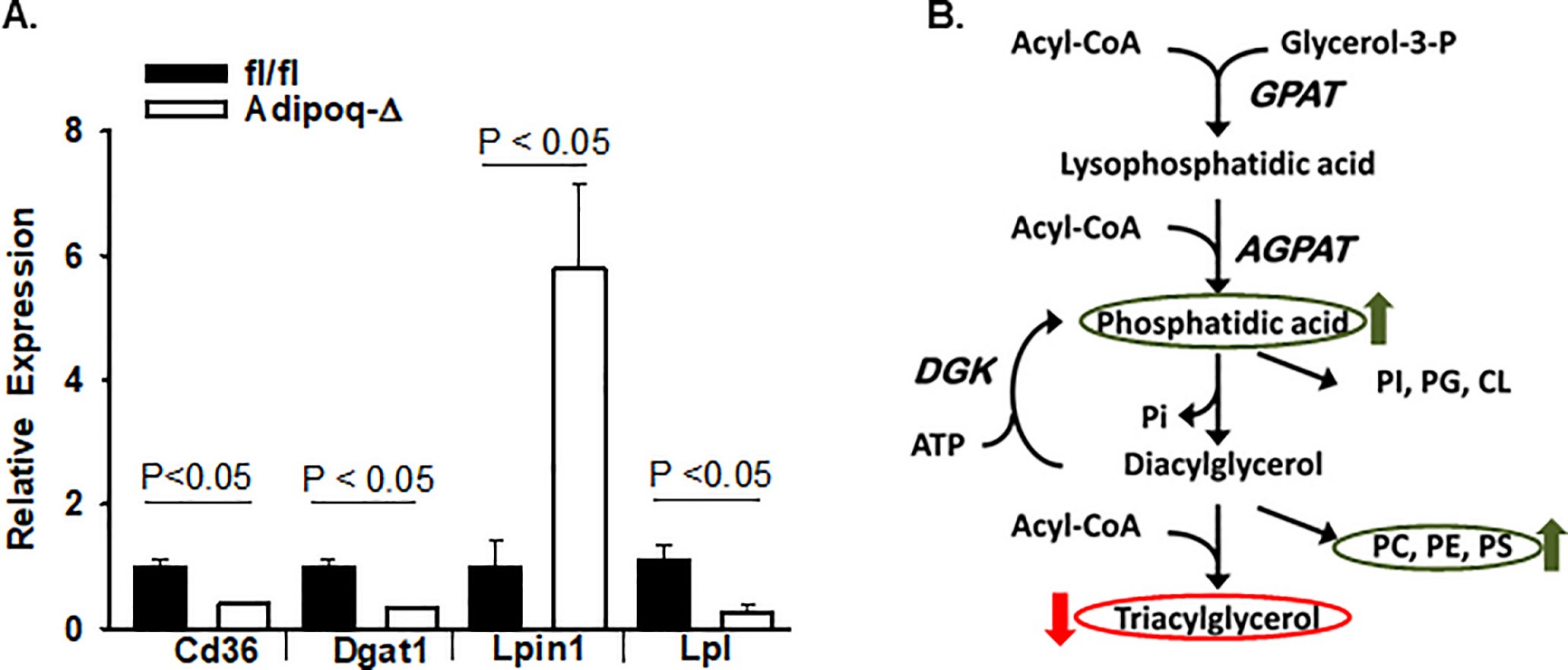
ATX regulates gene expression of enzymes that regulate phospholipid and triglyceride synthesis. **A.)** Gene expression in liver of fl/fl (dark bars) and Adipoq-Δ (open bars) male mice after 20 weeks on HFD (n = 3). **B.)** Summary of glycerophospholipids in liver influenced by ATX (arrows indicated changes in mice with reduced ATX expression) and pathways involved in their production.

Based on these results, we sought to determine if the loss of ATX altered expression of genes involved in fatty acid, diacylglcerol (DAG), PA and TG uptake/metabolism in the liver. For this analysis, we examined livers from Adipoq-Δ and fl/fl mice to avoid confounding effects of inflammatory changes observed in the MX1-Δ mice. Interesting, the adipose-specific knock-down mice expressed lower levels of *Cd36*, diacylglycerol acyltransferase (*Dgat1)*, and *Lpl* and higher levels of *Lipin1* in liver (Fig 8A), genes that regulate metabolic pathways as illustrated in Fig 8B.

## Discussion

Recent escalations in obesity have profound medical and socioeconomic implications.[33, 34] Excessive visceral fat deposition has been demonstrated to be strongly correlated with metabolic syndrome and cardiovascular diseases.[34] Moreover, the increased cardiovascular mortality in obese patients may not be completely explained by associated risk factors such as dyslipidemia (typically elevated TG and lower high density lipoproteins), hyperglycemia, insulin resistance and hypertension. Obesity is also characterized by a chronic sub-acute inflammatory state with macrophage infiltration in fat and the production of inflammatory mediators by adipose tissue [35]. In this work, we identify ATX as a mediator of the inflammatory response in the context of diet-induced obesity and importantly suggest that adipose-derived ATX a key regulator of lipid accumulation and/or remodeling that accompanies hepatic steatosis associated with obesity.

A comparison of the phenotypes of HFD-fed mice with global (MX1-Δ) and adipose-specific (Adipoq-Δ) reductions in ATX expression yield interesting similarities and differences following HFD feeding. In both models, the loss of ATX protected from diet-induced increases in adipose cell size and adipose inflammation. Likewise, mice displayed less HFD-associated accumulation of lipid in liver. Interesting, the global but not the adipose-specific deficiency had attenuated hepatic inflammation and were protected from insulin resistance. These observations could potentially reflect differences in local actions of ATX on liver inflammation (e.g. through resident macrophage or other cells) as plasma ATX was reduced by approximately 50% in both models. We did not address whether the effect of adipose ATX on liver lipid accumulation is a direct effect of adipose-derived ATX or indirect through changes in adipose inflammation.

ATX production can be stimulated by TNFα [36], likely in a nuclear factor-kappa B-dependent manner. Our results are consistent with a role for ATX in promoting local and systemic inflammation, the latter of which correlates with hepatic TG accumulation and glucose tolerance. The primary function of ATX is likely to generate bioactive LPA, which is known to promote inflammatory. Adipose-derived ATX predominately influences adipose inflammation, likely through local signaling effects, whereas broader effects of MX1-mediated global reduction in ATX were observed in both fat and liver tissue and translated into reductions in systemic, plasma levels of inflammatory mediators. The inflammatory changes that occur with obesity can in turn influence both insulin resistance and liver TG accumulation. For example, TNF-α over expression with obesity signals through IκB kinase β to phosphorylate IRS-1 and IRS-2 to promote insulin resistance [37, 38], which in turn promotes liver TG accumulation. Our observations of lower TNFα in mice with postnatal reductions in ATX may oppose this effect during HFD feeding. Likewise, the plasma insulin levels may promote adipose tissue lipolysis and reduce de novo liver synthesis. Although, interestingly, adipose-specific deletion of ATX altered liver lipids without a pronounced effect on liver inflammation.

Mice with global post-natal or adipose-specific deficiency of ATX were protected from accumulation of neutral lipids in the liver when fed high fat diet. Mass spectrometry based lipid profiling indicated that this phenotype was associated with steady state decreases in liver TG levels but significant increases in levels of several species of glycerophospholipids, including liver PC levels. The ratio PC:PE regulates membrane integrity, and decreases in the ratio elicits liver injury [39]. A significantly higher PC:PE ratio was observed in Adipoq-Δ mice, which lacked dramatic changes in liver inflammation that can impact lipid metabolism as described above. Results from liver RNA expression profiling in the suggest that alterations in adipose ATX levels are associated with changes in levels of expression of some key enzymes that catalyze critical steps that determine the substrate supply of phosphatidic acid and diacylglycerol for synthesis of phospholipids and TG, including *Cd36*, *Dgat1*, *Lipin1*, and *Lpl*. These findings could potentially explain the alterations in phospholipid accumulation in liver (Fig 8B). While genetic deficiency of ATX is protective against hepatic steatosis and reduces hepatic inflammation in mice, potent ATX inhibition had no effect on CCl4-induced liver fibrosis and choline-deficient amino acid-defined diet-induced liver injury in rats [40] and so it is possible that ATX does not contribute to liver dysfunction in these models.

LPA has a plethora of cellular effects that can regulate glucose metabolism and insulin signaling. It can directly inhibit islet cell secretion of insulin and increases glucose transport in muscle and fat [41]. LPA attenuates insulin signaling in the liver via LPAR3 by impairing PI3-kinase activation and glucokinase expression, the rate limiting enzyme in hepatic glucose utilization [42]. It is possible that the effects of global-reductions in ATX reflect these or other roles of LPA.

Our results indicate that while ATX has roles in regulating adipocyte size and inflammatory gene expression inducible systemic or adipocyte specific ATX deficiency has no effect on expansion of adipose depots during high fat feeding. Our finding that ATX regulates ectopic fat accumulation in the liver suggests that pharmacological inhibition of ATX might be effective in management of this growing complication of obesity and metabolic syndrome.

## Supporting information

S1 Table TaqMan gene expression (ThermoFisher) assays. Real-time quantitative PCR predesigned assays used to measure mRNA expression.
(PPTX)

S1 Fig Post-natal reductions in ATX expression with MX1-Cre mediated deletion. **A.)** Relative gene expression in different tissues in fl/fl (dark bars) and MX1-Δ (open bars) male mice (n = 6). **B.)** Immunoblot analysis of ATX protein in plasma (1 μl; bottom loading control = albumin) and plasma ATX activity (μmol/min/ml) in fl/fl (dark bars) and MX1-Δ (open bars) male mice (n = 6–7). **C.)** Immunoblot analysis of ATX protein expression in subcutaneous fat (loading control β-actin) and quantification of ATX expression in fat fl/fl (dark bars) and MX1-Δ (open bars) male mice.
(PPTX)

S2 Fig Effect of global post-natal reductions in ATX on liver gene expression. Relative gene expression in fl/fl (dark bars) and MX1-Δ (open bars) male mice (n = 3).
(PPTX)

S3 Fig Adipose-specific reductions in ATX expression. Relative gene expression in different tissues in fl/fl (dark bars) and Adipoq-Δ (open bars) male mice (n = 5–6). B.) ATX protein expression in subcutaneous and visceral fat. C.) Plasma ATX activity (μmols/min/ml) in fl/fl (dark bars) and Adipoq-Δ (open bars) male mice (n = 8). D. PPAR gene expression in subcutaneous adipose and (E) visceral adipose from fl/fl (dark bars) and Adipoq-Δ (open bars) male mice (n = 3).
(PPTX)

S4 Fig Effect of adipose-specific reductions in ATX on liver gene expression. Relative gene expression in fl/fl (dark bars) and Adipoq-Δ (open bars) male mice (n = 3).
(PPTX)
