# Supplementary material for: Adipose-derived autotaxin regulates inflammation and steatosis associated with diet-induced obesity
Source: PLoS One. 2019 Feb 7;14(2):e0208099. doi: 10.1371/journal.pone.0208099 (PMC6366870; doi:10.1371/journal.pone.0208099)
Supplement: S1 Table — Real-time quantitative PCR predesigned assays used to measure mRNA expression. (PPTX) [file pone.0208099.s001.pptx]

## Slide 1
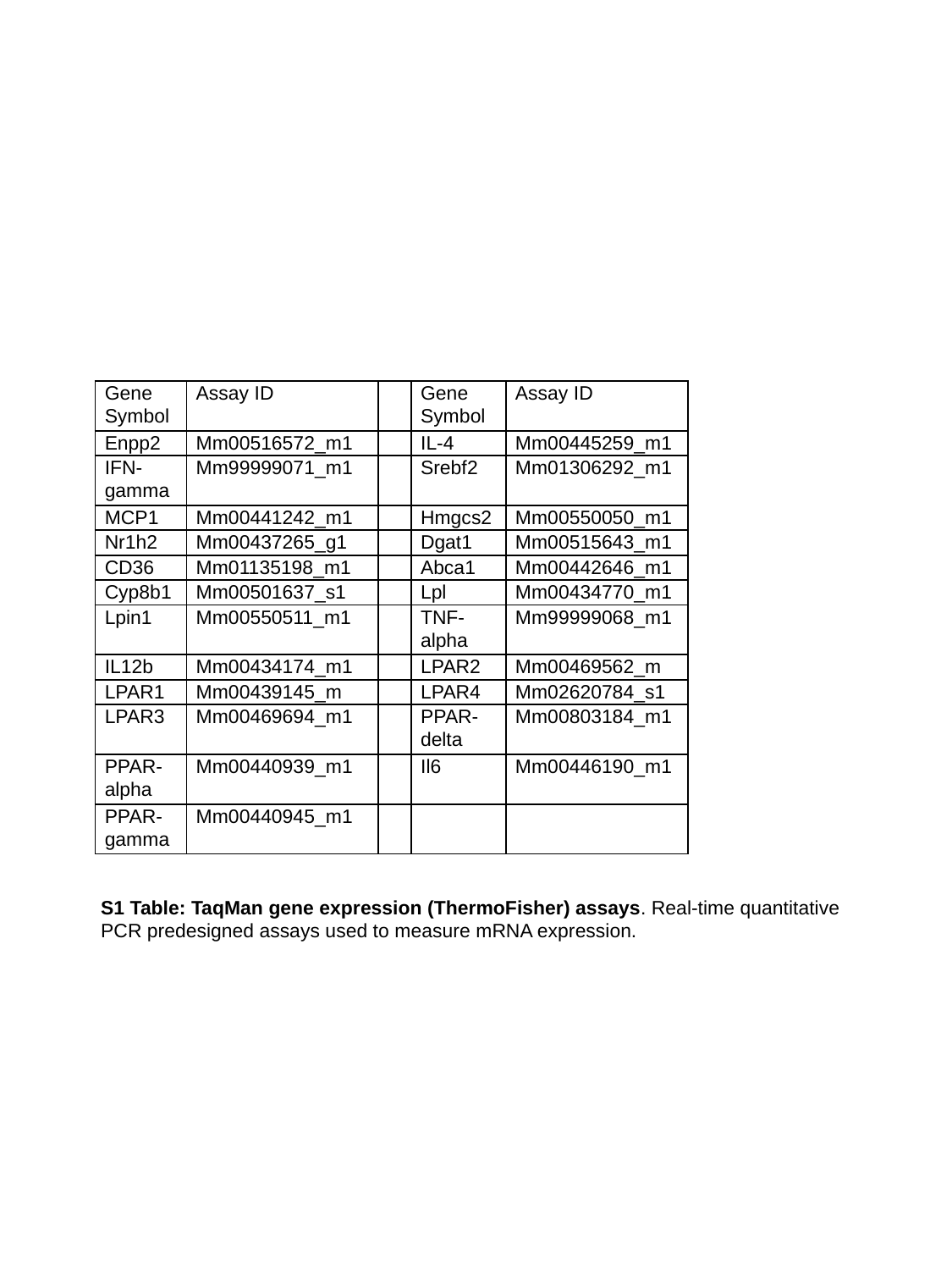

| Gene Symbol | Assay ID | | Gene Symbol | Assay ID |
| --- | --- | --- | --- | --- |
| Enpp2 | Mm00516572\_m1 | | IL-4 | Mm00445259\_m1 |
| IFN-gamma | Mm99999071\_m1 | | Srebf2 | Mm01306292\_m1 |
| MCP1 | Mm00441242\_m1 | | Hmgcs2 | Mm00550050\_m1 |
| Nr1h2 | Mm00437265\_g1 | | Dgat1 | Mm00515643\_m1 |
| CD36 | Mm01135198\_m1 | | Abca1 | Mm00442646\_m1 |
| Cyp8b1 | Mm00501637\_s1 | | Lpl | Mm00434770\_m1 |
| Lpin1 | Mm00550511\_m1 | | TNF-alpha | Mm99999068\_m1 |
| IL12b | Mm00434174\_m1 | | LPAR2 | Mm00469562\_m |
| LPAR1 | Mm00439145\_m | | LPAR4 | Mm02620784\_s1 |
| LPAR3 | Mm00469694\_m1 | | PPAR-delta | Mm00803184\_m1 |
| PPAR-alpha | Mm00440939\_m1 | | Il6 | Mm00446190\_m1 |
| PPAR-gamma | Mm00440945\_m1 | | | |
S1 Table: TaqMan gene expression (ThermoFisher) assays. Real-time quantitative
PCR predesigned assays used to measure mRNA expression.
