# Supplementary material for: Adipose-derived autotaxin regulates inflammation and steatosis associated with diet-induced obesity
Source: PLoS One. 2019 Feb 7;14(2):e0208099. doi: 10.1371/journal.pone.0208099 (PMC6366870; doi:10.1371/journal.pone.0208099)
Supplement: S1 Fig — A.) Relative gene expression in different tissues in fl/fl (dark bars) and MX1-Δ (open bars) male mice (n = 6). B.) Immunoblot analysis of ATX protein in plasma (1 μl; bottom loading control = albumin) and plasma ATX activity (μmol/min/ml) in fl/fl (dark bars) and MX1-Δ (open bars) male mice (n = 6–7). C.) Immunoblot analysis of ATX protein expression in subcutaneous fat (loading control β-actin) and quantification of ATX expression in fat fl/fl (dark bars) and MX1-Δ (open bars) male mice. (PPTX) [file pone.0208099.s002.pptx]

## Slide 1
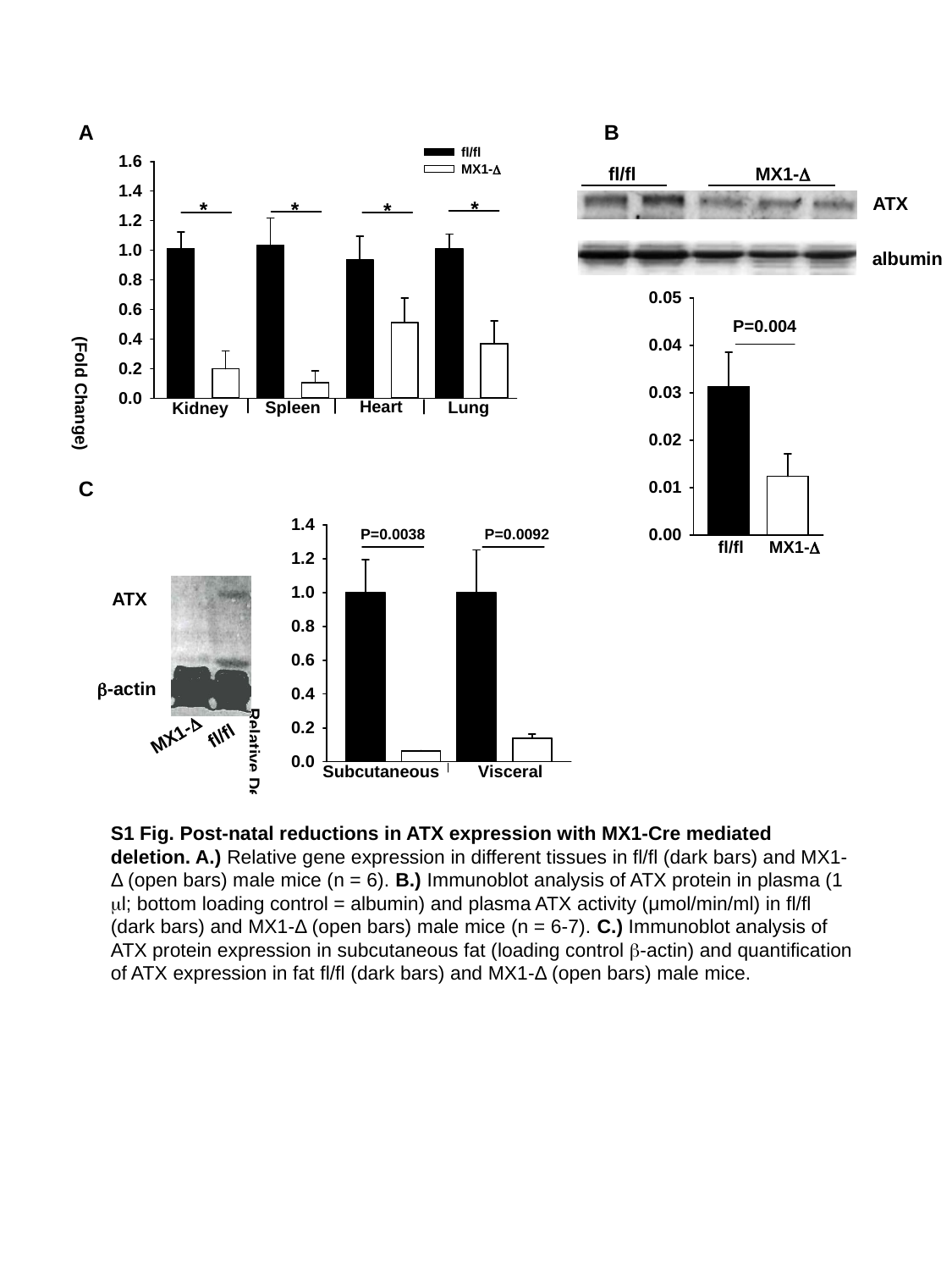

A
B
fl/fl
MX1-D
ATX
albumin
C
ATX
b-actin
MX1-D
fl/fl
S1 Fig. Post-natal reductions in ATX expression with MX1-Cre mediated deletion. A.) Relative gene expression in different tissues in fl/fl (dark bars) and MX1-Δ (open bars) male mice (n = 6). B.) Immunoblot analysis of ATX protein in plasma (1 ml; bottom loading control = albumin) and plasma ATX activity (μmol/min/ml) in fl/fl (dark bars) and MX1-Δ (open bars) male mice (n = 6-7). C.) Immunoblot analysis of ATX protein expression in subcutaneous fat (loading control b-actin) and quantification of ATX expression in fat fl/fl (dark bars) and MX1-Δ (open bars) male mice.
