# Supplementary material for: Adipose-derived autotaxin regulates inflammation and steatosis associated with diet-induced obesity
Source: PLoS One. 2019 Feb 7;14(2):e0208099. doi: 10.1371/journal.pone.0208099 (PMC6366870; doi:10.1371/journal.pone.0208099)
Supplement: S3 Fig — Relative gene expression in different tissues in fl/fl (dark bars) and Adipoq-Δ (open bars) male mice (n = 5–6). B.) ATX protein expression in subcutaneous and visceral fat. C.) Plasma ATX activity (μmols/min/ml) in fl/fl (dark bars) and Adipoq-Δ (open bars) male mice (n = 8). D. PPAR gene expression in subcutaneous adipose and (E) visceral adipose from fl/fl (dark bars) and Adipoq-Δ (open bars) male mice (n = 3). (PPTX) [file pone.0208099.s004.pptx]

## Slide 1
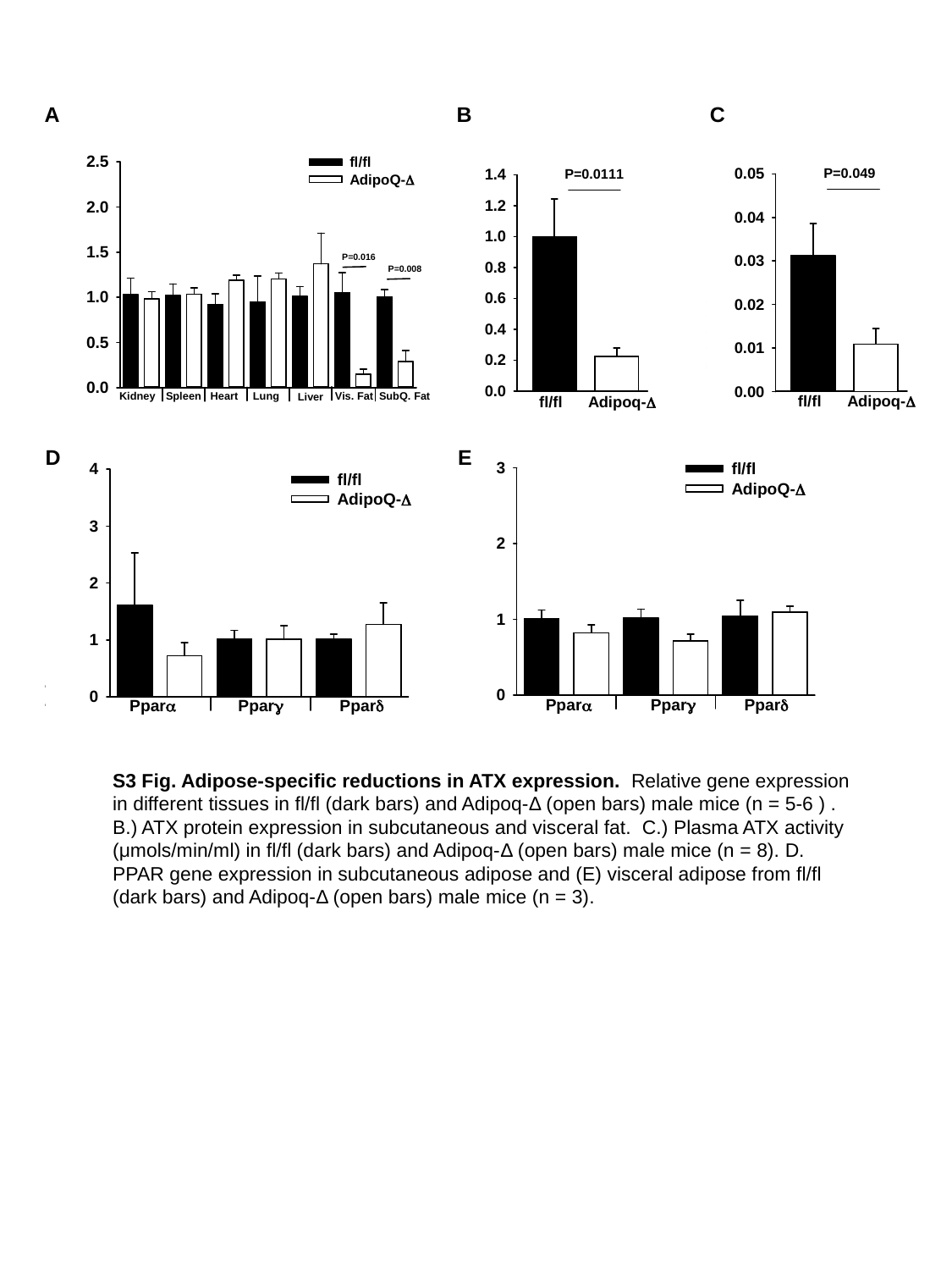

C
A
B
D
E
S3 Fig. Adipose-specific reductions in ATX expression. Relative gene expression in different tissues in fl/fl (dark bars) and Adipoq-Δ (open bars) male mice (n = 5-6 ) . B.) ATX protein expression in subcutaneous and visceral fat. C.) Plasma ATX activity (μmols/min/ml) in fl/fl (dark bars) and Adipoq-Δ (open bars) male mice (n = 8). D. PPAR gene expression in subcutaneous adipose and (E) visceral adipose from fl/fl (dark bars) and Adipoq-Δ (open bars) male mice (n = 3).
