# Supplementary material for: Adipose-derived autotaxin regulates inflammation and steatosis associated with diet-induced obesity
Source: PLoS One. 2019 Feb 7;14(2):e0208099. doi: 10.1371/journal.pone.0208099 (PMC6366870; doi:10.1371/journal.pone.0208099)
Supplement: S4 Fig — Relative gene expression in fl/fl (dark bars) and Adipoq-Δ (open bars) male mice (n = 3). (PPTX) [file pone.0208099.s005.pptx]

## Slide 1
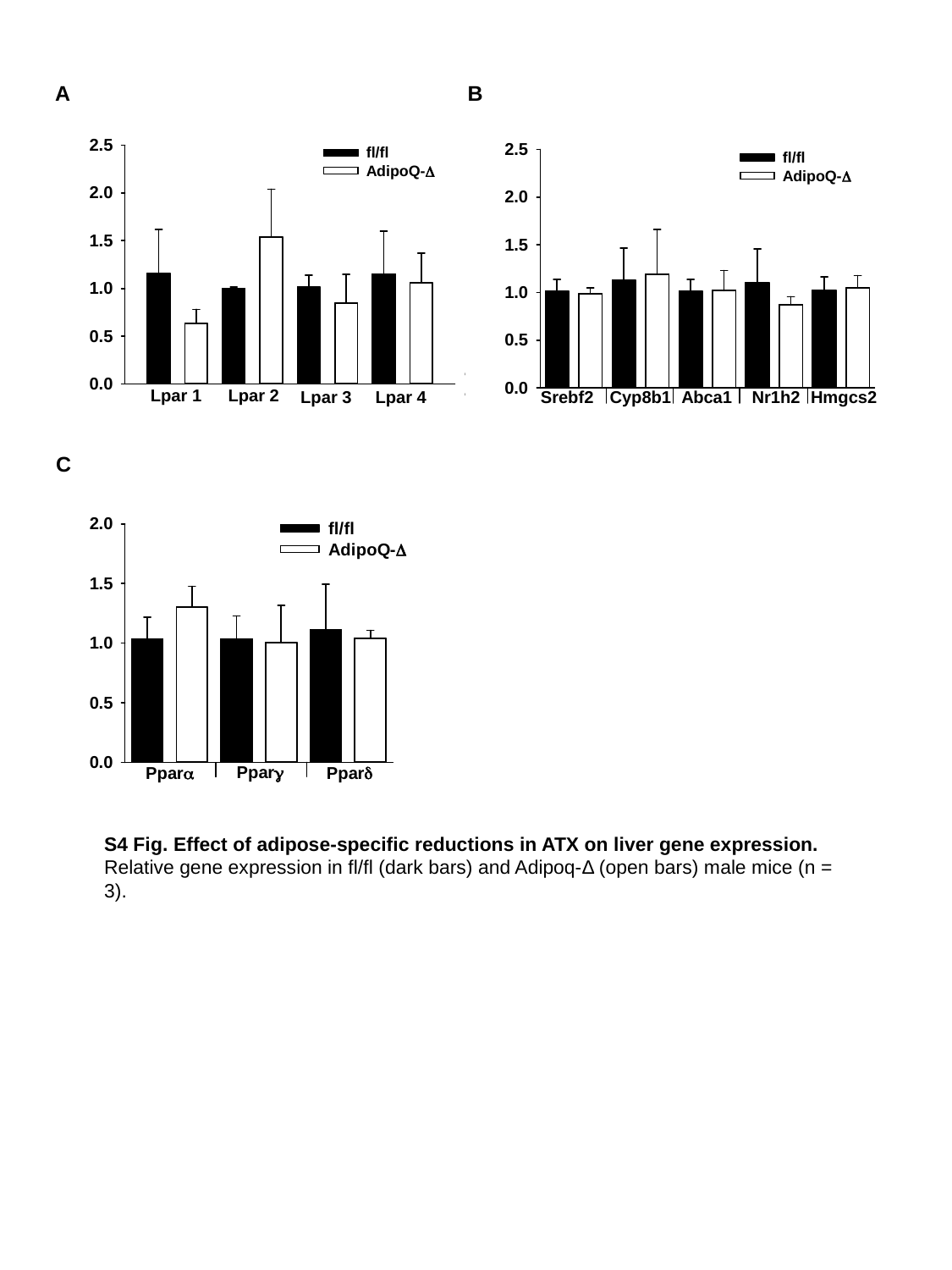

A
B
C
S4 Fig. Effect of adipose-specific reductions in ATX on liver gene expression. Relative gene expression in fl/fl (dark bars) and Adipoq-Δ (open bars) male mice (n = 3).
